# Effect of chemical modification of titanium dioxide surface with dicarboxylic acid on the crystalline parameters and rheology behavior in polypropylene composites.

**DOI:** 10.1016/j.dib.2018.08.141

**Published:** 2018-09-01

**Authors:** A. Almendarez-Camarillo, R. López-Esparza, R. Saldivar-Guerrero, J.A. Gonzalez-Calderon

**Affiliations:** a Departamento de Ingeniería Industrial, Posgrado en Ciencias en Ingeniería Bioquímica. Instituto Tecnológico de Celaya, Av. Tecnológico y Antonio García Cubas s/n. Celaya, Guanajuato 38010, Mexico; b Departamento de Física, 1626, Universidad de Sonora, Hermosillo, Sonora, 83000, Mexico; c Centro de Investigación en Química Aplicada, Blvd. Enrique Reyna Hermosillo 140, Saltillo, Coahuila 25294, Mexico; d Departamento de Ingeniería Química, Instituto Tecnológico de Celaya, Av. Tecnológico y Antonio García Cubas s/n. Celaya, Guanajuato 38010, Mexico

## Abstract

In this document, we present the effect of the surface modification of titanium dioxide particles with dicarboxylic acid on the rheological behavior of isotactic polypropylene composites. In addition to evaluating the effect of this type of modified fillers on the crystalline parameters such as long period, crystalline thickness and amorphous thickness, comparing it with unmodified fillers and pure polymer.

**Specifications table**

**Table:** 

Subject area	*Materials Science*
More specific subject area	*Polymer Composites*
Type of data	*Table and Figures*
How data was acquired	*Small Angle X-Ray Scattering* (Xeuss, whit Cu Kα radiation and a 2D dimension detector Pilatus 300 K)*, Rehometer with parallel plates* (TA Instruments, Model ARES G2).
Data format	*Analyzed*
Experimental factors	*Composites of iPP were obtained by hot press molding at 240 °C and immediately cooled at 20 °C. We use neat iPP, iPP filled with unmodified Titanium Dioxide particles with diameter of around 350 nm (n-TiO*_*2*_*) and iPP filled with modified titanium dioxide particles with pimelic acid with diameter of around 350 nm (c-TiO*_*2*_*). For both composites a concentration of 0.1% w/w of Titanium Dioxide were used.*
Experimental features	*All X-ray patterns were measured in air at room temperature.The specimens used were rectangular sheets measuring 2.5 mm in width, 0.5 mm in thickness and 22 mm in length.*
	*Rheology measurements were conducted at a temperature of 230° C using parallel plates of 25 mm diameter, with a gap of 1 mm. The corresponding standard is ASTM D-4440 for rheological properties of molten polymers.*
Data source location	*Not applicable*
Data accessibility	*Data are with this article*
Related research article	*Improvement in the energy dissipation capacity of polypropylene composites through a surface modification of titanium dioxide particles with a dicarboxylic acid. J.A. Gonzalez-Calderon, R. López-Esparza, R. Saldivar-Guerrero, A. Almendarez-Camarillo. Termochimica Acta, 2018, “Article in Press”*

**Value of the data**

● Importance of chemical modification of nanoparticles surface to change crystalline parameters.
● Complex viscosity behavior influenced by dicarboxylic acid linkage to nanoparticle surface.
● Chemical modification of titanium dioxide nanoparticles with dicarboxylic acids promotes lubricity of nanoparticles.

## 1 Data

Three samples were analyzed by means of the SAXS technique: iPP/c-TiO_2_, iPP/n-TiO_2_ and neat iPP. The lamellar stacking parameters were determined by means of the calculation of the correlation function in one dimension. The software is based on the fitting of the experimental curve to a theoretical model derived from the Fourier transformed of the distribution of the intensity for an ideal lamellar stacking. Table 1 shows the parameter values of the lamellar structure, amorphous thickness and the values of the long period, LP, obtained directly from the relation: *D* = 2Ѳ/*q*. We can observe that the addition of coated particles with pimelic acid reduces the Long period and the Crystalline thickness and increase the amorphous thickness in comparison with composites with unmodified particles, this is because of coating helps to integrate the iPP chains in a messed form [1]. This could be help to this kind of composites to dissipate in a better form the energy.

**Table 1:** Values of the lamellar structure, amorphous thickness and the values of the long period for the studied samples.

Sample	Long period [L_P_ nm]	Crystalline layers [L_C_ nm]	Amorphous layers [L_A_ nm]	*D* = 2π/*q*
iPP/n-TiO_2_	70.0	46.0	24.	69.5
iPP/c-TiO_2_	65.0	36.6	26.4	61.0
iPP	64.0	37.1	26.9	59.8

**Fig. 1:**
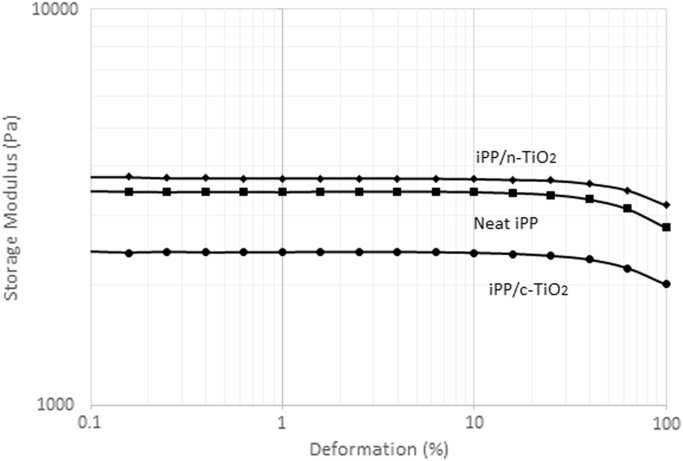
Deformation scans of the studied composites.

**Fig. 2:**
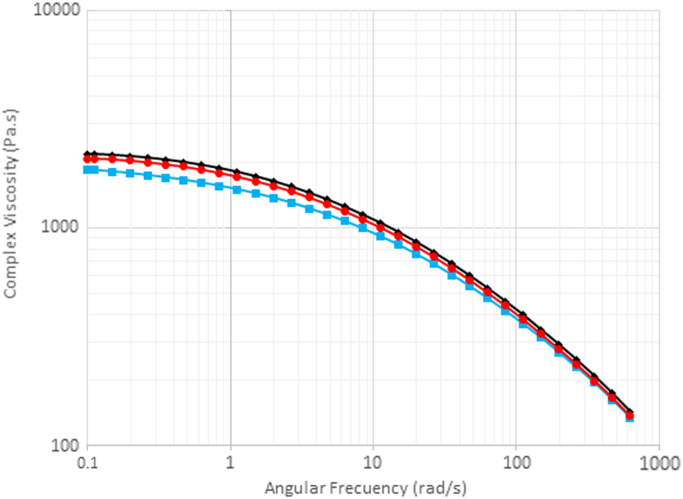
Complex viscosity versus angular frequency. Neat iPP (Black), and the composites with n-TiO_2_ particles (Red) and c-TiO_2_ particles (Blue) into iPP matrix.

Fig. 1 shows the deformation scans performed on the three samples analyzed. The strain selected to carry out the frequency sweeps was 10%. It can be observed that iPP/c-TiO_2_ composite shown a lower storage modulus, even that neat iPP, this experiment was conducted in molten state so we can observe that chemical modification of particles promotes the chain slide around them by the chemical affinity between pimelic acid molecules and the iPP chains. Fig. 2 shows the complex viscosity versus angular frequency curves for the three materials analyzed. With the incorporation of the n-TiO_2_ particles (Red) and c-TiO_2_ particles (Blue) into iPP matrix, an increase in the viscosity profile is observed in comparison both with the neat iPP (Black). This behavior is ascribed to the reinforcing effect of this filling particles [2].

## 2 Experimental design, materials, and methods

The method to modify the particles were provided by Gonzalez et al. [3], [4].

Composites of iPP were obtained by hot press molding at 240 °C and immediately cooled at 20 °C. We use neat iPP, iPP filled with unmodified Titanium Dioxide particles with diameter of around 350 nm (n-TiO_2_) and iPP filled with modified titanium dioxide particles with pimelic acid with diameter of around 350 nm (c-TiO_2_). For both composites a concentration of 0.1% w/w of Titanium Dioxide were used.

The oscillatory rheometry tests were carried out obtaining 25 mm diameter samples by means of a suction on 1 mm thick plates obtained by means of a hot compression press. One plate was obtained for each sample.

All X-ray patterns were measured in air at room temperature, using a high resolution SAXS equipment, trademark Xeuss, whit Cu Kα radiation (*α* = 0.1541 nm) and a 2D dimension detector (Pilatus 300 K). The specimens used were rectangular sheets measuring 2.5 mm in width, 0.5 mm in thickness and 22 mm in length.

The samples obtained were analyzed in an oscillating rheometer with the TA Instruments model ARES G2, at a temperature of 230 °C using parallel plates of 25 mm diameter, with a gap of 1 mm. The corresponding standard is ASTM D-4440 for rheological properties of molten polymers. The deformation was established at 10% after having performed a deformation sweep, and the frequency sweeps were carried out from 0.1 to 100 rad/s obtaining 25 points in the measurements (8 points per decade).
